# A Study on the Increase of Leakage Current in AlGaN Detectors with Increasing Al Composition

**DOI:** 10.3390/nano13030525

**Published:** 2023-01-28

**Authors:** Yujie Huang, Jing Yang, Degang Zhao, Yuheng Zhang, Zongshun Liu, Feng Liang, Ping Chen

**Affiliations:** 1 State Key Laboratory of Integrated Optoelectronics, Institute of Semiconductors, Chinese Academy of Sciences, Beijing 100083, China; 2 College of Materials Science and Optoelectronics Technology, University of Chinese Academy of Sciences, Beijing 100049, China; 3 Center of Materials Science and Optoelectronics Engineering, University of Chinese Academy of Sciences, Beijing 100049, China

**Keywords:** AlGaN, Schottky detectors, vacancy defect, dark leakage current

## Abstract

The dark leakage current of Al_x_Ga_1-x_N Schottky barrier detectors with different Al contents is investigated. It was found that the dark leakage of Al_x_Ga_1-x_N detectors increased with increasing Al content. The XRD and SIMS results showed that there was no significant difference of the dislocation density and carbon impurity concentration in five Al_x_Ga_1-x_N samples with different Al content. This was likely not the main reason for the difference in dark leakage current of Al_x_Ga_1-x_N detectors. However, the results of positron annihilation showed that the vacancy defect concentration increased with increasing Al content. This was consistent with the result that the dark leakage current increased with increasing Al content. With the increase of vacancy concentration, the vacancy defect energy levels also increased, and the probability of electron tunneling through defect levels increased. In contrast, the Schottky barrier height decreased, which eventually led to the increase of dark leakage current. This discovery should be beneficial to an accurate control of the performance of Al_x_Ga_1-x_N detectors.

## 1. Introduction

Since Al_x_Ga_1-x_N ultraviolet (UV) detectors can play an important role in flame warning, biochemical detection, missile tracing, biomedical detection and other areas involving UV detection [1,2,3], there has been much research carried out on Al_x_Ga_1-x_N UV photodetectors in recent years. Several studies have been carried out on various types of detectors, such as Schottky barrier [4], Metal–Semiconductor–Metal (MSM) [5] and p-i-n [6] structures. In addition, solar-blind focused plane arrays [7] have been realized. Among all kinds of structures, the Schottky barrier detector is relatively simple in structure and has the advantages of short response time and high quantum efficiency [8,9]. However, the dark current of Schottky detectors is larger. So far, the dark leakage mechanism of Al_x_Ga_1-x_N Schottky detectors is still not very clear. Many researchers have proposed that dislocations are the source of leakage [10,11]. In addition, some scholars have studied Al_x_Ga_1-x_N detectors with different Al content in the past, and the results showed that the concentration of vacancy increased significantly with increased Al content [12]. However, until now, there was no evidence to prove whether the presence of vacancy really affects the dark leakage current of the detector. Therefore, it is necessary to study the relationship between point defects and dark leakage current of photodetectors, which should be beneficial for the preparation of high-performance AlGaN ultraviolet Schottky detectors.

This work focuses on the dark leakage current characteristics of Al_x_Ga_1-x_N Schottky barrier UV detectors with different Al contents. The results showed that the dark leakage current of Al_x_Ga_1-x_N Schottky detectors increased with increased Al content. The mechanism may be that the increase of Al content leads to the increase of concentration of vacancy defects, and these defects greatly increase the probability of tunneling current and decrease the Schottky barrier height. Therefore, reducing the vacancy defect concentration in the material is very important to reduce the dark leak current of Al_x_Ga_1–x_N Schottky detectors.

## 2. Experiment

**Figure 1:**
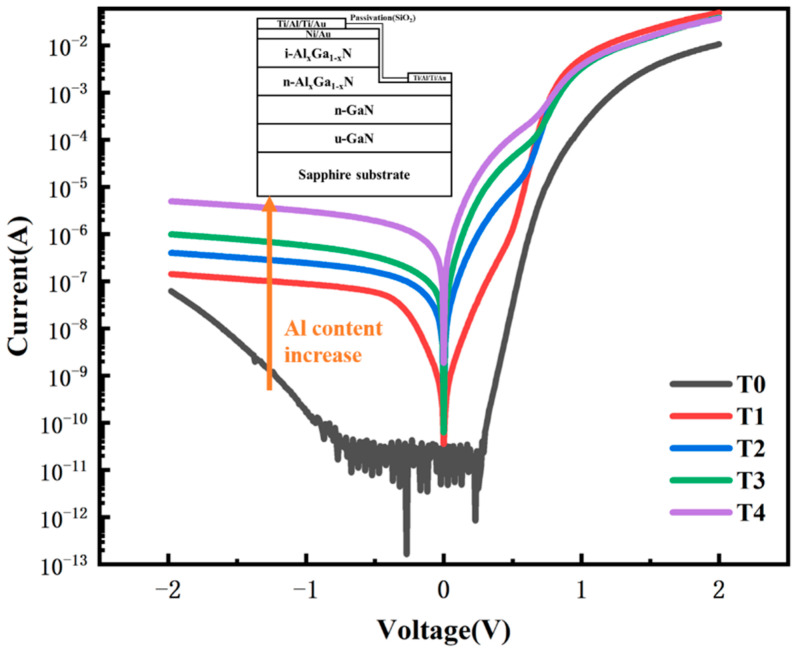
I–V curves of Al_x_Ga_1-x_N Schottky barrier ultraviolet photodetector samples T0–T4.Tthe schematic diagram of the Schottky diode devices is in the inset.

Five Al_x_Ga_1-x_N (x < 7%) Schottky barrier ultraviolet photodetectors were prepared during this study for comparison. They had nearly the same device structure, but the Al content of the AlGaN active layers was different. The epilayers were grown on c-plane sapphire substrate by metalorganic chemical vapor deposition (MOCVD). These photodetectors used the metal–Al_x_Ga_1-x_N Schottky contact structure, of which the schematic diagram is shown in the inset of Figure 1. During the material growth, firstly a GaN buffer layer was grown on the sapphire substrate, followed by a 1.7 um n-GaN layer, a 500 nm n-type n-Al_x_Ga_1-x_N layer and a 250 nm unintentionally doped i-Al_x_Ga_1-x_N layer. The five samples had Al_x_Ga_1-x_N active layers of different Al composition. The different Al contents were obtained by adjusting the Trimethyl Aluminum (TMAl) flow rate. The TMAl flux employed during the growth of Al_x_Ga_1-x_N layers was 0 umol/min, 2 umol/min, 3 umol/min, 4.5 umol/min, and 6 umol/min for samples T0–T4, respectively. The detectors devices were then fabricated using traditional semiconductor technology. Photolithography and ion beam etching were used to define the shape of the device. The contact electrodes of the devices were fabricated using the electron beam evaporation and lift-off technique. The top transparent Schottky barrier was formed on the i- Al_x_Ga_1-x_N layer by using a Ni/Au (10nm/10nm) metal film, and the bottom Ohmic contact to the i- Al_x_Ga_1-x_N layer was made using Ti/Al/Ti/Au metal. 

The full width at half maximum (FWHM) measurements of the high-resolution X-ray diffraction (002) and (102) Omega scan were performed on five samples using the Rigaku SmartLab X-Ray Diffractometer. The surface morphology of i-AlGaN layer was analyzed by employing the Bruker Dimension Icon atomic force micrometer (AFM) in tapping mode with OTESPA-R3 tips. The surface roughness of samples was analyzed by NanoScope Analysis 1.8. In addition, the impurity concentration distribution in the samples was measured by secondary ion mass spectroscopy (SIMS), and the photoluminescence (PL) spectra of AlGaN detector samples were measured by grating spectrometer using 325 nm a He-Cd laser as the excitation source. Moreover, positron annihilation was used to measure the vacancy defect concentrations of samples. The positron source used was ^22^Na, and the positron energy incident on the sample was continuously adjusted. Furthermore, the current–voltage (I–V) characteristics were measured by a Keithley 2400 source meter. All measurements were carried out at room temperature.

## 3. Results and Discussion

Figure 1 shows the I–V curves of samples T0–T4 measured at bias scanning from −2V to +2V. Firstly, the forward current in each I–V curve rose rapidly after an opening voltage, which indicated typical behavior of the rectifier characteristics of Schottky junctions. Actually, the I–V curves of five samples had different shapes. Most importantly, a remarkable difference was observed between the reverse currents of the five curves. As shown in Table 1, it was noticed that the obtained values of reversed currents were $6.22\times 10^{-8}A$, $1.42\times 10^{-7}A$, $4.02\times 10^{-7}A$, $9.87\times 10^{-7}A$, $\mathrm{and}\;4.96\times 10^{-6}A$ at a reverse bias voltage of −2 V for samples T0–T4, respectively. Furthermore, the Schottky barrier height was calculated according to the formula of I–V characteristics [13]. The values were 1.0 eV, 0.9 eV, 0.8 eV, 0.7 eV, and 0.7 eV for samples T0–T4, respectively, while the same metal and the same surface treatment processing were adopted for Schottky contacts of all samples. It was noted that the dark leakage current increased with the increase of Al content. 

**Figure 2:**
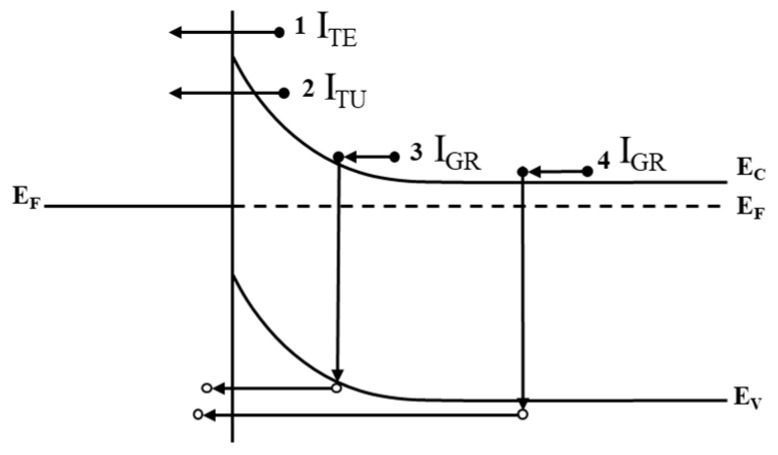
Transport processes under 0 bias. The processes number 1–number 4 are thermionic emission (1 I_TE_), tunneling current (2 I_TU_), generation-recombination current (3 I_GR_) in space charge region, and hole injection from the metal to the semiconductor (equivalent to generation-recombination current in neutral region, 4 I_GR_). (E_F_ is fermi level, E_C_ is Conduction band level and E_V_ is Valence band level.)

The current transport in metal-semiconductor contacts is mainly due to majority carriers. There are several carrier transport mechanisms in Schottky junctions: thermionic emission, field emission, tunneling, and generation recombination current, as schematically shown in Figure 2 [14]. First, thermionic emission refers to the thermal emission of the majority carriers in a semiconductor over the barrier into the metal. Second, since trap levels are distributed in the space charge region, this can greatly increase the probability for electrons to tunnel from the semiconductor side to the metal side. According to the tunneling model, the bending of the band becomes the barrier for carriers to tunnel to the interface state or defect. There may be multiple tunneling caused by multiple trap levels. Next, generation-recombination in the depletion region is another mechanism that affects the transmission of current through the barrier. Furthermore, the recombination of holes injected from the metal into the semiconductor in the neutral region also affects current transport. The reverse leakage current density is composed of leakage current components formed by various mechanisms. Abnormal leakage current may occur when tunneling current accounts for a large part. In addition, there are many defects in the material itself. The larger the component of some defects, the larger the leakage current will be. 

Many research reports have dealt with the leakage current of GaN devices. A growing number of groups provides evidence that the dislocations in the group-III nitrides behave as non-radiative recombination centers, and may provide a leakage current pathway [15,16]. However, in this work something different was observed. The FWHMs of the ω-scan rocking curves measured by HRXRD, which are closely related to the edge and screw dislocation densities, are listed in Table 1. The (002) FWHM values of T0–T4 samples were 291, 298, 293, 284, and 275 arc sec, respectively; and the (102) FWHM values of T0–T4 samples were 310, 306, 301, 304, and 310 arc sec, respectively. They were nearly the same in these samples. In general, there was no significant difference in the dislocation densities of the five samples, so the dislocations were not the reason for the change of dark leakage of the detectors with different Al contents. Therefore, we assumed there must something else that was causing the leakage current to be different, and so we performed a series of other experiments. 

**Figure 3:**
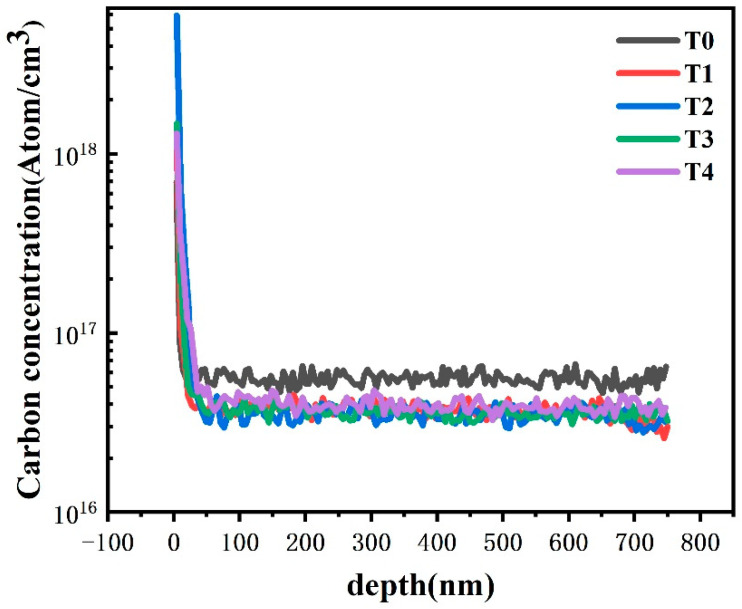
The carbon concentration distribution in the five samples T0–T4.

Most of these previous studies involved the role of dislocation in the leakage current. It was believed that the carbon impurity concentration contamination could lead to an increased leakage current and carbon impurity contamination; thus, it was proposed they could lead to an increase of leakage current [17]. However, the carbon impurity concentration of the 5 AlGaN Al_x_Ga_1−x_N layers measured by SIMS (Figure 3), showed that there was almost no significant, remarkable difference in the concentration of carbon impurities in samples T1–T4. Due to the large Ga flux during the growth of the GaN sample T0, the SIMS results showed that the carbon concentration of sample T0 was slightly higher. However, the leakage current of sample T0 was the lowest among the five samples, which indicates that the carbon impurity should not be the essential reason for the increase of the leakage current of samples T1–T4. 

In summary, according to the SIMS and XRD results, neither dislocation nor carbon impurity concentration was the essential reason affecting the dark leakage current of the five samples. Therefore, it can be suggested that other factors may play a more important role in enhancing the dark leakage current of Al_x_Ga_1–x_N photodetectors than dislocations and carbon impurities. We proposed it was due to some other reasons, such as some kinds of point defects in the material [18].

**Figure 4:**
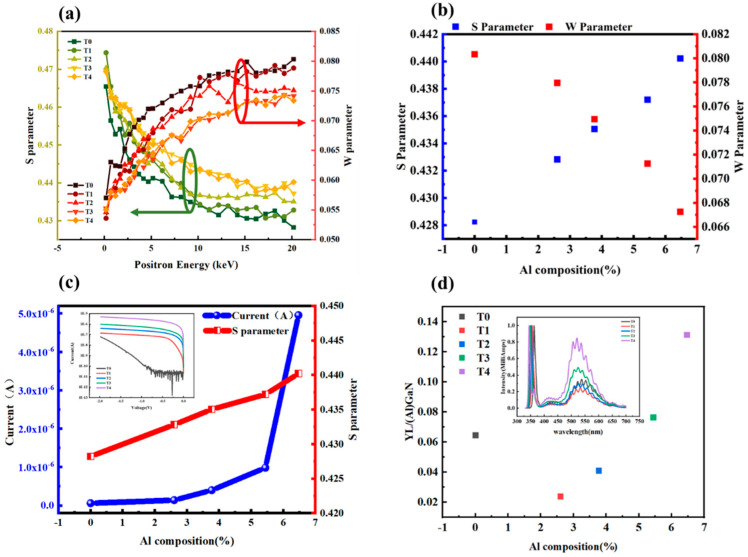
(**a**) The dependence of low momentum parameter S and high momentum parameter Won positron incident energy in the five samples. (**b**) The Al composition dependences of S parameter and W parameter. (**c**) Reverse current at the -2V bias (blue) and S parameter (red) versus Al composition in AlGaN; the inset is reverse I–V curves of samples T0–T4. (**d**) Al composition dependence of S parameter and W parameter; the inset is PL spectra of five samples T0–T4.

Positron annihilation technology can effectively study point defects in materials, especially vacancy type defects such as cationic vacancy defects and vacancy group. When there are defects in the material, the positrons freely diffused in the material are captured by vacancy defects [19]. Due to the lack of positive ions in the vacancy, the positrons are less likely to annihilate the inner core electrons and mainly annihilate the surrounding valence electrons, resulting in a narrower Doppler broadening [19,20]. Therefore, these point defects can be detected by detecting the Doppler broadening of annihilation radiation [21]. In order to discern point defects in Al_x_Ga_1-x_N photodetectors, positron annihilation experiments were employed. In these experiments, the Doppler broadening spectra of the 511 keV annihilation radiation were recorded using conventional instrumentation. The Al_x_Ga_1−x_N layers were investigated with Doppler broadening experiments using a variable energy positron beam, the energy of which was chosen so that all positron could annihilate in the Al_x_Ga_1−x_N epilayer. The shape of the 511 keV line was described using the conventional low and high electron momentum parameters S and W. Parameter S was defined as the ratio of the count in the central region of the peak to the total number of peaks in the Doppler broadening spectrum of 511 keV, reflecting the annihilation of positron–electron pairs in the low momentum region; parameter W was defined as the ratio of the count on the two sides of the peak to the total number of peaks, reflecting the annihilation of positron–electron pairs in the high momentum region. When positrons annihilate at vacancies, the S parameter increases and the W parameter decreases as a larger faction of annihilations takes place with the low momentum valence electrons [19]. The Figure 4a shows the S parameter and W parameter each as a function of positron incident energy for the five samples. As can be seen from Figure 4a,b, with increased Al content, the S parameter curve rose upward and the W parameter curves downward from sample T0 to T4. The increase of the S parameter and the decrease of the W parameter indicate that the position capture probability of the material increased; that is, the vacancy defect concentration increased [22]. Therefore, it can be seen from Figure 4a,b that the vacancy defect concentration in the Al_x_Ga_1−x_N epitaxial wafer increased with increased Al content. Using the slope of the S–W curve, the type of defects in the samples could be judged. When S–W parameters fell on the same line, it indicated that the types of defects were consistent. The results showed that the S–W parameter curves of different samples were basically coincident, indicating that these samples had similar cationic vacancy defect structures. This result indicated that there was only one kind of vacancy point defect in all five samples. Such vacancy defects may be assigned to Ga vacancies (V_Ga_) or its complexes [19]. 

In fact, our experimental results were consistent with the reported theoretical model which suggests that during the MOCVD growth, the Ga vacancies are less likely to form under Ga-rich conditions [23,24]. When the flow rate of Al decreases and flow rate of Ga increases, the formation of Ga vacancy may be suppressed, thus the concentration of Ga vacancy in sample T0 was the lowest. Ga vacancy formation energy of MOCVD-AlGaN is low under N-rich conditions. It is found in the literature that the O substitute at N site (O_N_) and Ga vacancy (V_Ga_) constitute complex defects, and it is confirmed that the positron in AlGaN samples is mainly captured by V_Ga_ complexes. When positrons annihilate in defects, an increase of the number of defects will result in increased Doppler broadening in the high momentum region and decreased in the low momentum region; that is, the W parameter increases and the S parameter decreases. With the increase of Al flow rate, the content of the Al component in the sample increases, the band gap width of the Al_x_Ga_1-x_N sample increases, the relative position of Fermi level changes, and the formation energy of Ga vacancy decreases. The formation energy of Ga vacancy in Al_x_Ga_1−x_N is less than that of Ga vacancy in GaN, so as Al composition increases, the Ga vacancy concentration increases.

Figure 4d shows the comparison of PL intensity of the Al_x_Ga_1-x_N peak and yellow luminescence band (YL) of the five samples at room temperature. It was noted that the intensity of YL was closely related with vacancy concentration, and the PL result provided more favorable evidence that the vacancy concentration in the five samples was significantly correlated with the Al component of Al_x_Ga_1−x_N. Figure 4d shows that with the increased Al component, the YL intensity showed an upward trend (where sample T0 may have had higher YL intensity due to the slightly higher concentration of carbon impurities in T0). It is known that the origin of YL is related to both carbon impurities [25] and vacancy defects [26]. However, the results of the SIMS proved that there was no significant difference in the concentration of carbon impurities in samples T1–T4. Meanwhile, the results of positron annihilation indicated that the vacancy defects concentration increased with the increased Al component. The results of Figure 4 were consistent with those of Figure 4d. Therefore, the YL results further confirm that the concentration of vacancy defects in Al_x_Ga_1−x_N increase with the increase of Al content. This may be an important reason for the obvious increase in the dark leakage current of the five detectors.

Point defects can form defect levels, through which electrons can tunnel to form a tunneling current. On the one hand, the higher the vacancy concentration, the deeper energy levels may be formed, and the probability of electron tunneling will also increase. These vacancy defects may form a continuum of states within the forbidden band on which leakage electrons can move. Electrons from the contact metal could overcome the locally reduced Schottky barrier and tunnel onto the vacancy-related continuum of states through thermionic field emission. On the other hand, due to the fact that the same metal and the same surface treating processing are adopted, the effective barrier height is mainly related to the vacancy concentration [27]. With the increase of vacancy concentration, the effective barrier height of the Schottky barrier decreases, which may be also one of the reasons for the increased leakage current. Both of these two factors may lead to an increase in the tunneling current, resulting in an increase in the reverse leakage current of the Schottky detector. In addition, besides the suggested dominant dark leakage mechanism, other minor or parasitic leakage mechanisms could co-exist in the reverse transport process. Meanwhile, the existence of tunneling current directly affects the dominant carrier transport mode in the Schottky detector under forward voltage, thus leading to different shapes of the I–V curves observed in Figure 1.

The Ga vacancies are regarded as deep level centers in Al_x_Ga_1–x_N. Recently it has been found that the minority carrier diffusion length decreases by the effect of Ga vacancies in GaN due to the influence of such deep level defects [28]. Therefore, it is thought that the increase of deep level defects formed by vacancy defects can also lead to an increase in tunneling current probability. Figure 4c shows the relationship between Al content, S parameter and dark leakage current at a bias of –2V. With the increase of Al content, vacancy concentration increases and dark leakage current increases. It is worth emphasizing that an increase in Al composition has a promotive influence on the vacancy concentration and dark leakage current, but they do not necessarily have a one-to-one linear correspondence. 

## 4. Conclusions

The dark leakage current of Al_x_Ga_1−x_N(x < 7%) Schottky barrier ultraviolet photodetectors was investigated. It was found that the Al_x_Ga_1−x_N photodetector with increasing Al composition up to nearly 7% exhibited increased dark leakage current. The results of HRXRD and SIMS showed that there was no significant difference in the dislocation density and carbon impurity concentration among the five studied samples, indicating that they were not the essential reason affecting the dark leakage current of the Al_x_Ga_1−x_N photodetector. However, the results of positron annihilation strongly proved that the vacancy defect concentration increases with increased Al composition. Meanwhile, the results of PL provide evidence for the existence of vacancy defects. On the one hand, the increased vacancy concentration may form more defect levels to increase the probability of the tunneling current. On the other hand, the effective barrier height of Schottky contacts decreases due to the increase of the vacancy concentration. The combined action of these two aspects may increase the leakage current of the detectors with higher Al composition.

## Figures and Tables

**Table 1:** The characterization parameters of the T0–T4 samples.

Samples	FWHM of HRXRD (arc sec)		Screw Dislocation Density (em^−2^)	Edge Dislocation Density (cm^−2^)	Dark Leakage Current at −2 V (A)
	(002)	(102)			
T0	291	310	$1.70\times 10^{8}$	$5.10\times 10^{8}$	$6.22\times 10^{-8}$
T1	298	306	$1.78\times 10^{8}$	$4.97\times 10^{8}$	$1.42\times 10^{-7}$
T2	283	301	$1.61\times 10^{8}$	$4.81\times 10^{8}$	$4.02\times 10^{-7}$
T3	284	304	$1.62\times 10^{8}$	$4.91\times 10^{8}$	$9.87\times 10^{-7}$
T4	275	310	$1.52\times 10^{8}$	$5.10\times 10^{8}$	$4.96\times 10^{-6}$

## Data Availability

The data that support the findings of this study are available from the corresponding author upon reasonable request.
